# Supplementary material for: ATP activates bestrophin ion channels through direct interaction
Source: Nat Commun. 2018 Aug 7;9:3126. doi: 10.1038/s41467-018-05616-4 (PMC6081419; doi:10.1038/s41467-018-05616-4)
Supplement: Supplementary file 1 — Supplementary Information [file 41467_2018_5616_MOESM1_ESM.pdf]

Supplementary Information

ATP Activates Bestrophin Ion Channels through Direct Interaction

Zhang et al.

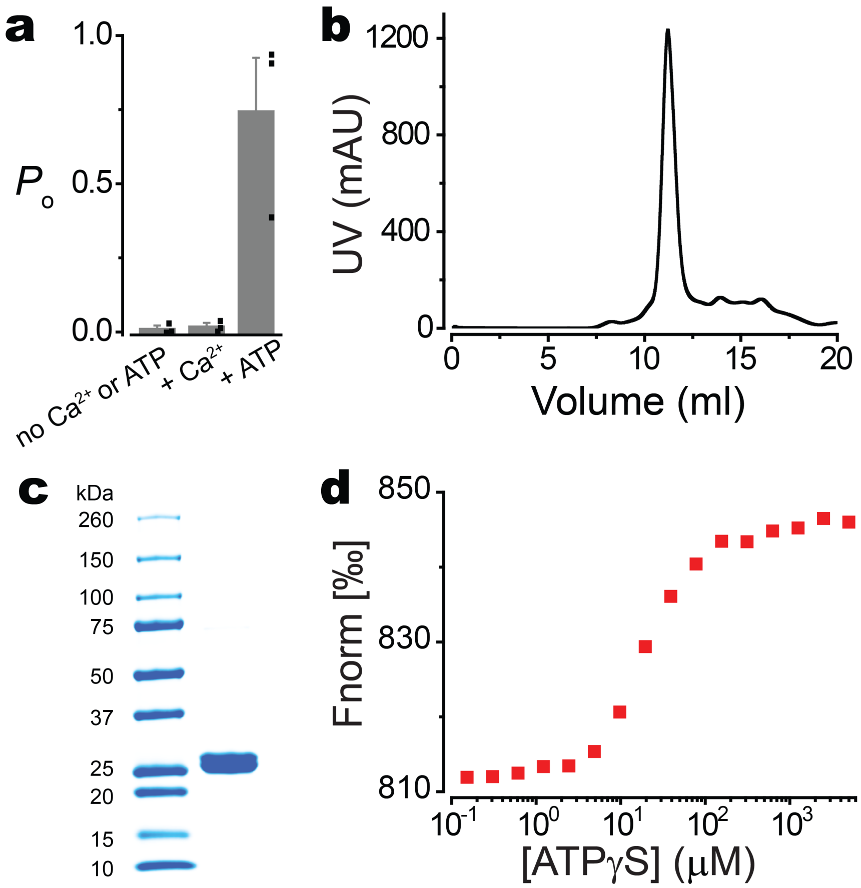

Supplementary Figure 1 The properties of purified KpBest
(**a**) Open probabilities of KpBest in the presence of 10 mM EGTA: with neither Ca^{2+} nor ATP, with 1 μM free Ca^{2+}, or with 2mM ATP; n= 3. (**b**) Representative size exclusion profile of purified KpBest protein. (**c**) Representative SDS-PAGE gel of purified KpBest protein. (**d**) An exemplar F_{norm} data of ATPγS to purified KpBest in MST. All error bars in this figure represent s.e.m.

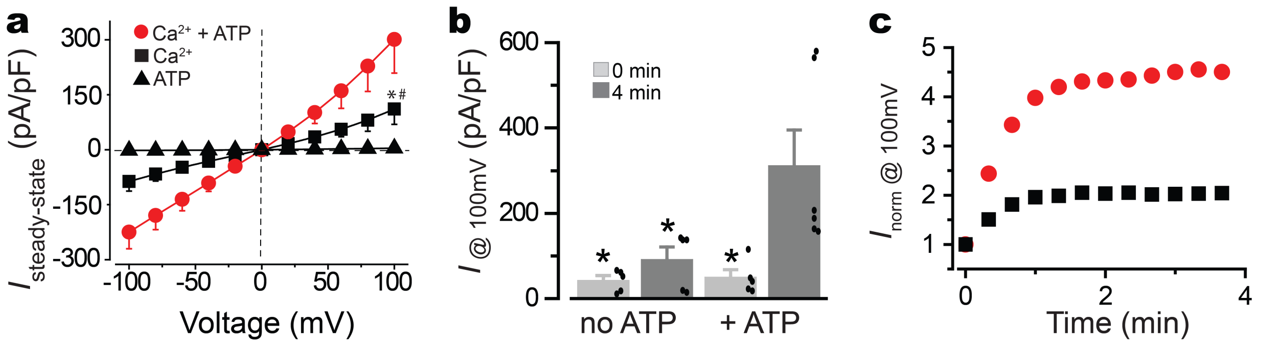

Supplementary Figure 2 The influence of ATP on Ca^{2+}-dependent Cl^- current in human RPE cells

(**a**) Population steady-state current-voltage relationships in WT iPSC-RPE in the presence of ATP (10 mM) without Ca^{2+}, Ca^{2+} (0.6 μM) without ATP, or both Ca^{2+} (0.6 μM) and ATP (10 mM), n= 5-6 for each point. *#$P < 0.05$ compared to ATP only and Ca^{2+} + ATP, respectively, using two-tailed unpaired Student *t* test. (**b**) Bar chart showing time-dependent activation in the presence of Ca^{2+} (0.6 μM) without ATP, or both Ca^{2+} (0.6 μM) and ATP (10 mM); n= 5-6 for each bar. *$P < 0.05$ compared to 4 min in the presence of ATP, using one-way ANOVA and Bonferroni *post hoc* analyses. (**c**) Time-dependent activation of representative RPE surface Cl^- current amplitudes in the presence of Ca^{2+} (0.6 μM) without (black) or with (red) ATP (10 mM). All error bars in this figure represent s.e.m.

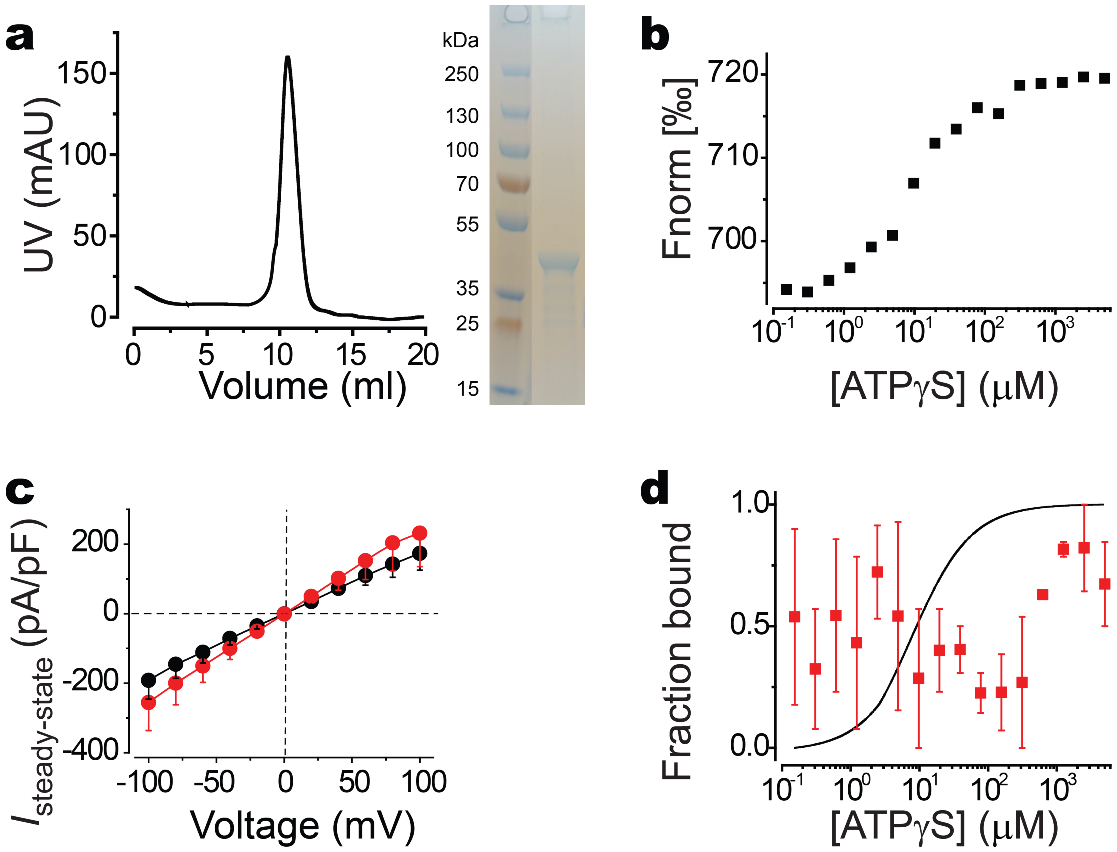

Supplementary Figure 3 ATP interacts with WT bBest2 but not the I201T mutant
(**a**) *Left*, purified bBest2 ran on a size exclusion gel-filtration column as one main peak; *right*, purified bBest2 on a SDS-PAGE gel. (**b**) Exemplar F_{norm} data of ATPγS binding to purified bBest2 in MST. (**c**) Population steady-state current-voltage relationships of transiently expressed bBest2 I201T in HEK293 cells without (black) or with (red) ATP (10 mM), n= 5-6 for each point. (**d**) MST data points of ATPγS binding to bBest2 I201T (red) compared to the binding curve of ATPγS to WT bBest2 (black). n= 3. All error bars in this figure represent s.e.m.

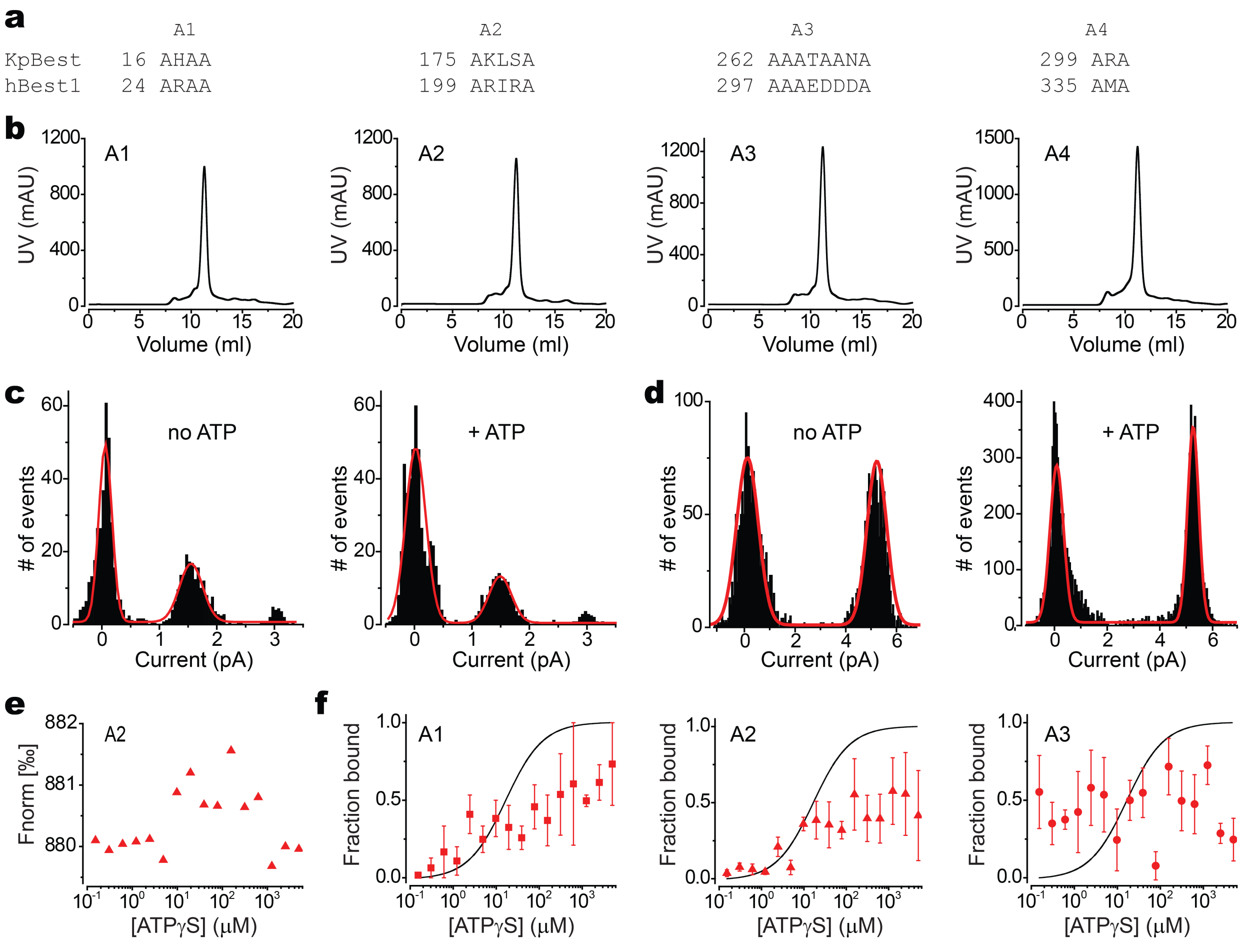

Supplementary Figure 4 KpBest A2 mutant is defective in ATP binding and ATP-dependent activation

(**a**) A1-A4 mutants of KpBest and hBest1. Numbers indicate the position of the first residue. (**b**) Representative size exclusion profiles for KpBest A1-A4 mutants. (**c, d**) Histograms showing single channel current amplitudes of KpBest A2 (**c**) or A4 (**d**) in the absence and presence of 2 mM ATP. (**e**) *E*xemplar F_{norm} data of ATPγS binding to purified KpBest A2 in MST. (**f**) MST data points of ATPγS binding to KpBest A1-A3 (red) compared to the binding curve of ATPγS to WT KpBest (black). n= 3 for each point. All error bars in this figure represent s.e.m.

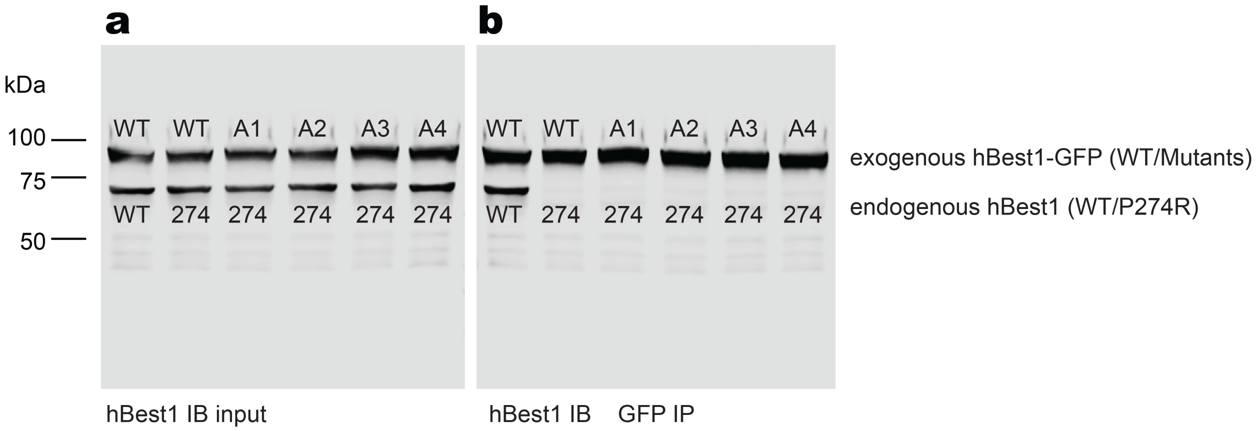

Supplementary Figure 5 Endogenous hBest1 P274R mutant cannot interfere with the assembly of the pentameric WT/A1-A4 channels

(**a, b**) Exogenous hBest1-GFP and endogenous hBest1 were detected by immunoblotting in input (**a**) and co-immunoprecipitation (**b**) samples.

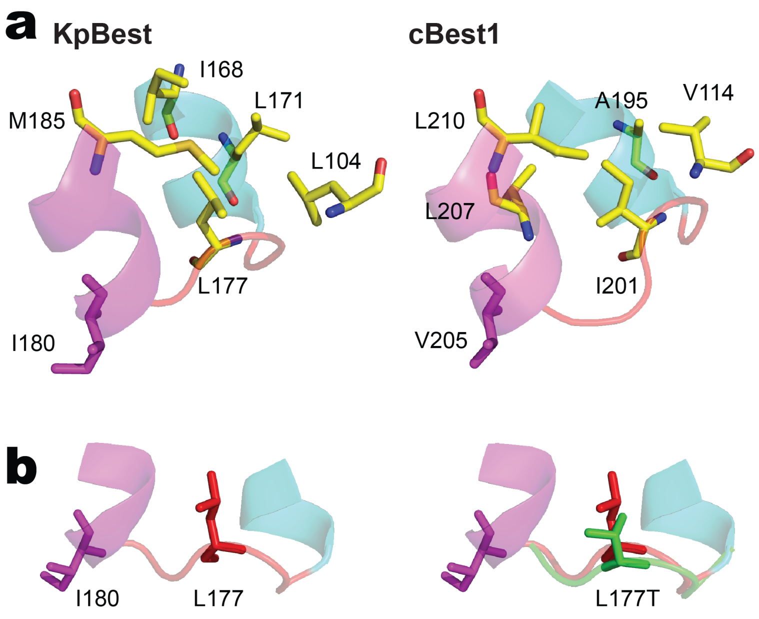

Supplementary Figure 6 The ATP-binding loop and neighboring hydrophobic residues in bestrophin structures

(**a**) Visualization of the ATP-binding loop (red) and all five surrounding hydrophobic residues. *Left*, KpBest; *right*, cBest1. (**b**) Visualization of the shift of the ATP-binding loop caused by the KpBest L177T mutation. *Left*, WT KpBest; *right*, superposition of WT KpBest (red) and the L177T mutant loop (green).

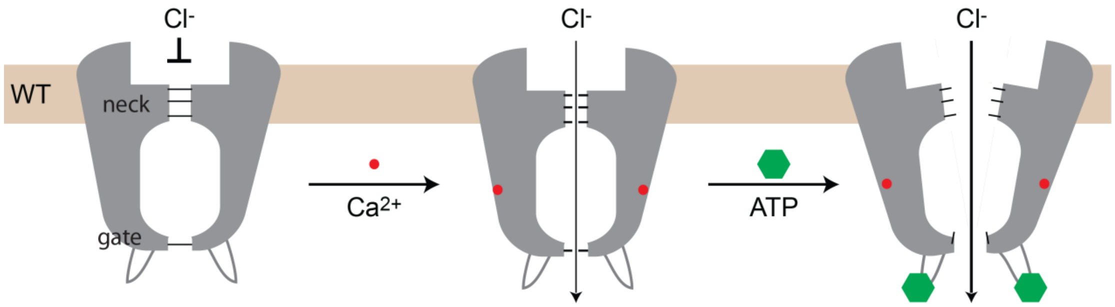

Supplementary Figure 7 A two-step activation model of hBest1
Cartoon showing the activation of hBest1 by Ca^{2+} and ATP.

Supplementary Table 1 Comparison of different data sets from the same donors

	0 mM ATP		2 mM ATP	
Clone	#1	#2	#1	#2
WT (pA pF^{-1})	98.2 ± 32.1	87.3 ± 25.8	316.2 ± 105.4	258.5 ± 77.3
I201T (pA pF^{-1})	38.8 ± 19.7	44.7 ± 20.2	49.6 ± 18.8	58.0 ± 25.6

Ca^{2+}-dependent Cl^- current amplitudes in two clonal iPSC-RPEs (for WT and I201T). n= 5-6 for each data set. Data are presented as means ± s.e.m.

Supplementary Table 2 Radii at critical residues in the ion conducting pathway of bestrophins

kpBest		cBest1	
Residue	Radius (Å)	Residue	Radius (Å)
I62	1.3	I76	1.1
I66	2.2	F80	1.4
F70	2.3	F84	2.3
I180	0.9	V205	1.3

Radius was calculated based on measuring the distances between five CH_3- (C delta on isoleucine or valine, a1-a5) or H- (benzene ring on phenylalanine, b1-b5):

For isoleucine or valine, radius = (a1+a2+a3+a4+a5) / (5*1.17) – 2.00 (van der Waals radius of CH_3-)
For phenylalanine, radius = (b1+b2+b3+b4+b5) / (5*1.17) – 1.20 (van der Waals radius of H-)

Supplementary Table 3 Key resource

REAGENT or RESOURCE	SOURCE	IDENTIFIER
Antibodies		
BESTROPHIN1	Novus Biologicals	NB300-164
Alexa Fluor 555-conjugated IgG	Invitrogen, Life Technologies	A-21422
Goat anti-mouse IgG, Dylight 800	ThermoFisher	SA5-35521
GFP	Invitrogen, Life Technologies	A6455
Bacterial and Virus Strains		
BacMam-hBest1 WT	Laboratory of Tingting Yang	N/A
BacMam-hBest1 A1	This paper	N/A
BacMam-hBest1 A2	This paper	N/A
BacMam-hBest1 A3	This paper	N/A
BacMam-hBest1 A4	This paper	N/A
BacMam-bBest2 (1-406)	This paper	N/A
DH10Bac	New York Structural Biology Center	N/A
DH5alpha	Laboratory of Wayne Hendrickson	N/A
BL21 plysS	Laboratory of Wayne Hendrickson	N/A
Biological Samples		
Chemicals, Peptides, and Recombinant Proteins		
KpBest A1	This paper	N/A
KpBest A2	This paper	N/A
KpBest A3	This paper	N/A
KpBest A4	This paper	N/A
bBest2 (1-406)	This paper	N/A
bBest2 (1-406) I201T	This paper	N/A
Critical Commercial Assays		
In-fusion HD Cloning Kit	Clontech	639645
Experimental Models: Cell Lines		
HEK293-F cells	New York Structural Biology Center	N/A
HEK293 cells	Laboratory of David Yule	N/A
iPSC-RPE (BEST1 WT)	Laboratory of Stephen H Tsang	N/A
iPSC-RPE (BEST1 P274R)	Laboratory of Stephen H Tsang	N/A
iPSC-RPE (BEST1 I201T)	Laboratory of Stephen H Tsang	N/A
Experimental Models: Organisms/Strains		
Oligonucleotides		

Primers for cloning bBest2 (1-406)<br>Forward:<br>TCACTAGTCGCGGCCGCATGACCGTC<br>ACGTACACGGC<br>Reverse:<br>GAAGTAGAGGTTCTCTGTGCCCATGC<br>CCGT	This paper	N/A
Primers for making Kpbest A1<br>Forward:<br>GCGCACGCCGCCGTGCTGTCGAAAAT<br>CATC<br>Reverse:<br>CACGGCGGCGTGCGCGTCAAATAGG<br>CGAAGAAACCAGTG	This paper	N/A
Primers for making Kpbest A2<br>Forward:<br>AAGCTCAGCGCCATCACTTACGGGCT<br>GATGG<br>Reverse:<br>GATGGCGCTGAGCTTGGCCGCCTCC<br>CGCAGC	This paper	N/A
Primers for making Kpbest A3<br>Forward:<br>ACCGCAGCCAATGCCCTGCCGTTGAA<br>CGCGA<br>Reverse:<br>GGCATTGGCTGCGGTGGCGGCCGCA<br>TCTTCCAGCTCTTCCGCCA	This paper	N/A
Primers for making Kpbest A4<br>Forward:<br>GCCTTCAATCTGACCTGACATTGGAA<br>GTGG<br>Reverse:<br>GGTCAGATTGAAGGCACGGGCCGGA<br>CGCAGCGTCT	This paper	N/A
Primers for making hBest1 A1<br>Forward:<br>GCGCGGGCCGCCATCTACAAGCTGCT<br>ATATGGCGAGT<br>Reverse:<br>GATGGCGGCCCGCGCGCACAGCAGC<br>AGGCGG	This paper	N/A
Primers for making hBest1 A2<br>Forward:<br>GCCCCTATCCTGCTCCAGAGCC<br>Reverse:<br>GAGCAGGATAGGGGCCCGGATTCGA<br>GCTCCAAGCCACGCCTTCA	This paper	N/A

Primers for making hBest1 A3<br>Forward:<br>GCAGAGGATGATGATGCTTTTGAGAC<br>CAACTGGATTGTCGACAG<br>Reverse:<br>ATCATCATCCTCTGCAGCGGCGTTGA<br>TGAGCTGCTCTGCCA	This paper	N/A
Primers for making hBest1 A4<br>Forward:<br>GCCTGGAATAAGCCCGAGCCAC<br>Reverse:<br>GGGCTTATTCCAGGCCATGGCCGGCT<br>CCATCCGAGG	This paper	N/A
Primers for cloning bBest2 I201T<br>Forward: ACCCGCGACAACGGCG<br>Reverse:<br>GCCGTTGTCGCGGGTGCGGCCCTCG<br>CG	This paper	N/A
Recombinant DNA		
pMCSG7-10xHis-KpBest A1	This paper	N/A
pMCSG7-10xHis-KpBest A2	This paper	N/A
pMCSG7-10xHis-KpBest A3	This paper	N/A
pMCSG7-10xHis-KpBest A4	This paper	N/A
pEG BacMam-hBest1 A1-GFP-10xHis	This paper	N/A
pEG BacMam-hBest1 A2-GFP-10xHis	This paper	N/A
pEG BacMam-hBest1 A3-GFP-10xHis	This paper	N/A
pEG BacMam-hBest1 A4-GFP-10xHis	This paper	N/A
pEG BacMam-bBest2 (1-406)-GFP-10xHis	This paper	N/A
pEG BacMam-bBest2 (1-406) I201T-GFP-10xHis	This paper	N/A
